# Supplementary material for: Efficacy and Safety of Thermal Ablation after Endoscopic Mucosal Resection: A Systematic Review and Network Meta-Analysis
Source: J Clin Med. 2024 Feb 25;13(5):1298. doi: 10.3390/jcm13051298 (PMC10932371; doi:10.3390/jcm13051298)
Supplement: Supplementary file 1 [file jcm-13-01298-s001.zip › jcm-2838130-supplementary.pdf]

Supplementary Table S1. Embase search strategy.

No.	Query	Results
#1	endoscopic mucosal resection*' OR 'emr' OR 'endoscopic aspiration mucosectom*' OR 'endoscopic mucosa resection*' OR 'endoscopic mucosectom*' OR 'endoscopic mucous membrane resection*' OR 'endoscopic mucosal resection'/syn	32874
#2	'argon plasma coagulation'/syn OR 'argon plasma coagulat*' OR 'argon beam coagulat*' OR 'apc' OR 'ar plasma*' OR 'argon plasma*'	62805
#3	'snare tip soft coagulation'/syn OR 'snare tip soft coagulat*' OR 'stsc'	161
#4	#2 OR #3	62937
#5	#1 AND #4	1125
#6	colon*:ti,ab,kw,de OR colorect*:ti,ab,kw,de OR rectum*:ti,ab,kw,de OR rectal*:ti,ab,kw,de OR 'colorectal polyp'/syn OR 'colorectal adenoma'/syn	1454272
#7	#5 AND #6	540
#8	#7 NOT ([animals]/lim NOT [humans]/lim) NOT ('conference review'/it OR 'editorial'/it OR 'letter'/it OR 'note'/it OR 'review'/it OR 'short survey'/it OR 'tombstone'/it OR 'case report'/de OR 'meta analysis'/de OR 'meta analysis topic'/de OR 'systematic review'/de OR 'systematic review topic'/de)	394

Supplementary Table S2. Risk of bias in non-randomized studies of interventions (ROBINS-I) scale for illustration of risk of bias of studies.

Study	Confounding	Selection	Intervention Classification	Deviation from Intervention	Missing Data	Measurement of Outcome	Selection of Reported Results	Total
Abu Arisha 2023	Low	Low	Low	Low	Low	Low	Low	Low
Groff 2020	Low	Low	Low	Low	Low	Low	Low	Low
Kandel 2019	Low	Low	Low	Low	Low	Low	Low	Low
Katsinelos 2019	Low	Low	Low	Low	Low	Low	Low	Low
Levenick 2022	Low	Low	Low	Low	Low	Low	Low	Low
Nader 2022	Low	Low	Low	Low	Low	Low	Low	Low
Perez 2021 (1)	Low	Low	Low	Low	Low	Low	Low	Low
Perez 2021 (2)	Low	Low	Low	Low	Low	Low	Low	Low
Wehbeh 2020	Low	Low	Low	Low	Low	Low	Low	Low

Supplementary Table S3. Risk of bias table of randomized controlled trials using Cochrane risk of bias tool.

Study	Random Sequence Generation (Selection bias)	Allocation Concealment (Selection Bias)	Blinding of Participants and Personnel (Performance Bias)	Blinding of Outcome Assessment (Detection Bias)	Incomplete Outcome Data (Attrition Bias)	Selective Reporting (Reporting Bias)
Albuqurque 2013	Unclear	Low	High	Unclear	Low	Low
Brooker 2002	Unclear	Low	High	Unclear	Low	Low
Klein 2019	Low	Low	High	High	Low	Low
Rex 2023	Low	Low	High	High	Low	Low
Senada 2020	Unclear	Low	High	Unclear	Low	Low

Supplementary Table S4. Sensitivity analysis of network meta-analysis using netsplit technique.

Comparison	K	Prop	Nma	Direct	Indir.	Diff	Z	p-Value
Recurrence								
APC:placebo	5	0.74	-1.12	-0.98	-1.51	0.53	0.90	0.3666
APC:STSC	2	0.53	0.21	0.18	0.23	-0.05	-0.09	0.9274
STSC:placebo	9	0.97	-1.32	-1.35	-0.70	-0.64	-0.98	0.3288
Total adverse events								
APC:placebo	3	0.60	-0.63	-0.69	-0.53	-0.16	-0.28	0.7772
APC:STSC	2	0.74	-0.09	-0.08	-0.11	0.03	0.05	0.9622
STSC:placebo	3	0.95	-0.54	-0.51	-1.02	0.51	0.73	0.4657

k: Number of studies providing direct evidence, prop: Direct evidence proportion, nma: Estimated treatment effect (logOR) in network meta-analysis, direct: Estimated treatment effect (logOR)

derived from direct evidence, indir.: Estimated treatment effect (logOR) derived from indirect evidence, Diff: Difference between direct and indirect treatment estimates, z: z-value of test for disagreement (direct versus indirect) (APC: argon plasma coagulation, STSC: snare tip soft coagulation).

A.

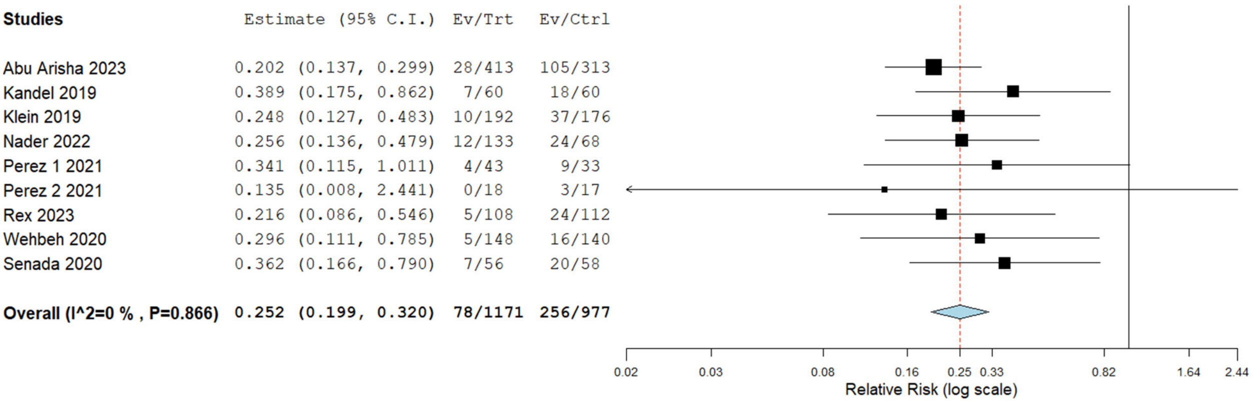
Studies
Estimate (95% C.I.) Ev/Trt Ev/Ctrl
Abu Arisha 2023 0.202 (0.137, 0.299) 28/413 105/313
Kandel 2019 0.389 (0.175, 0.862) 7/60 18/60
Klein 2019 0.248 (0.127, 0.483) 10/192 37/176
Nader 2022 0.256 (0.136, 0.479) 12/133 24/68
Perez 1 2021 0.341 (0.115, 1.011) 4/43 9/33
Perez 2 2021 0.135 (0.008, 2.441) 0/18 3/17
Rex 2023 0.216 (0.086, 0.546) 5/108 24/112
Wehbeh 2020 0.296 (0.111, 0.785) 5/148 16/140
Senada 2020 0.362 (0.166, 0.790) 7/56 20/58
Overall (I^2=0 % , P=0.866) 0.252 (0.199, 0.320) 78/1171 256/977
0.02 0.03 0.08 0.16 0.25 0.33 0.82 1.64 2.44
Relative Risk (log scale)

B.

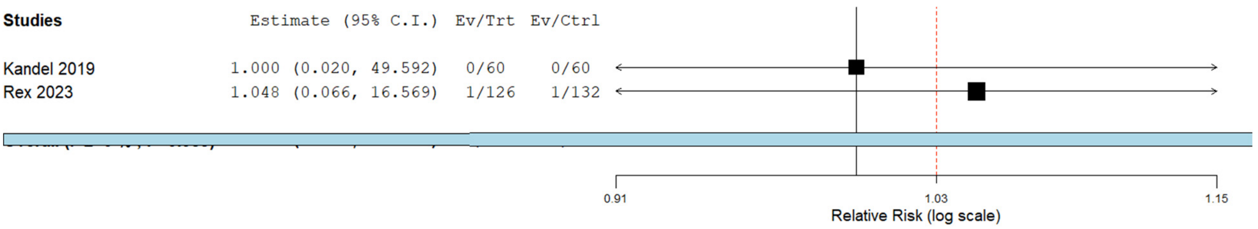
Studies
Estimate (95% C.I.) Ev/Trt Ev/Ctrl
Kandel 2019 1.000 (0.020, 49.592) 0/60 0/60
Rex 2023 1.048 (0.066, 16.569) 1/126 1/132
0.91 1.03 1.15
Relative Risk (log scale)

C.

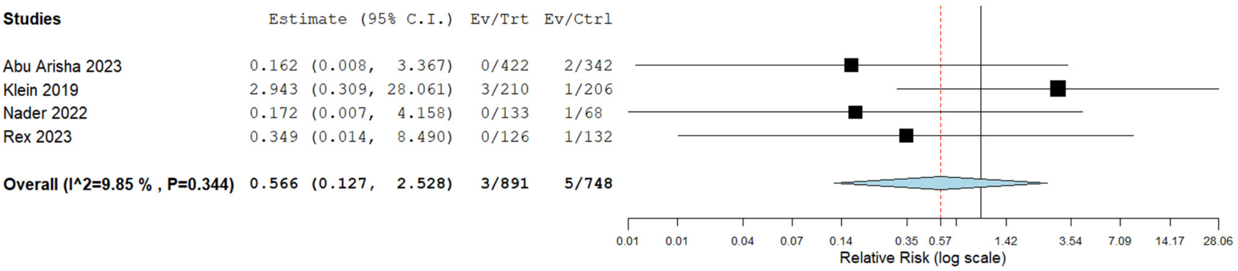
Studies
Estimate (95% C.I.) Ev/Trt Ev/Ctrl
Abu Arisha 2023 0.162 (0.008, 3.367) 0/422 2/342
Klein 2019 2.943 (0.309, 28.061) 3/210 1/206
Nader 2022 0.172 (0.007, 4.158) 0/133 1/68
Rex 2023 0.349 (0.014, 8.490) 0/126 1/132
Overall (I^2=9.85 % , P=0.344) 0.566 (0.127, 2.528) 3/891 5/748
0.01 0.01 0.04 0.07 0.14 0.35 0.57 1.42 3.54 7.09 14.17 28.06
Relative Risk (log scale)

D.

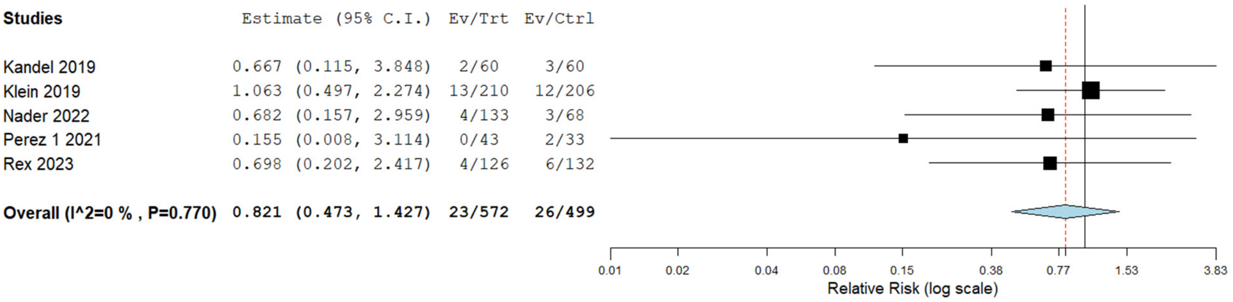

E.

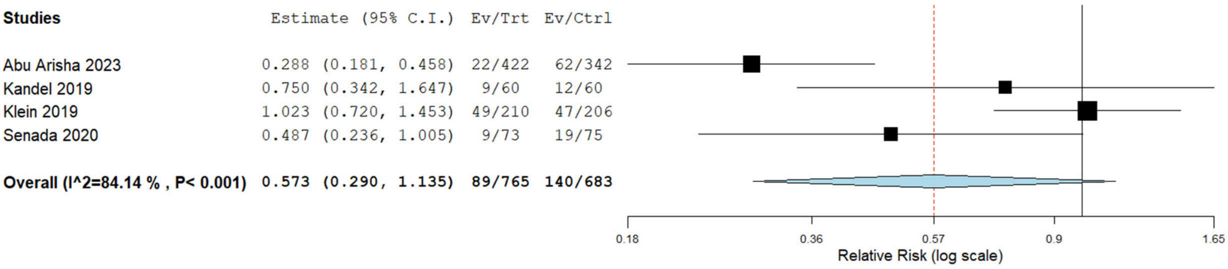

F.

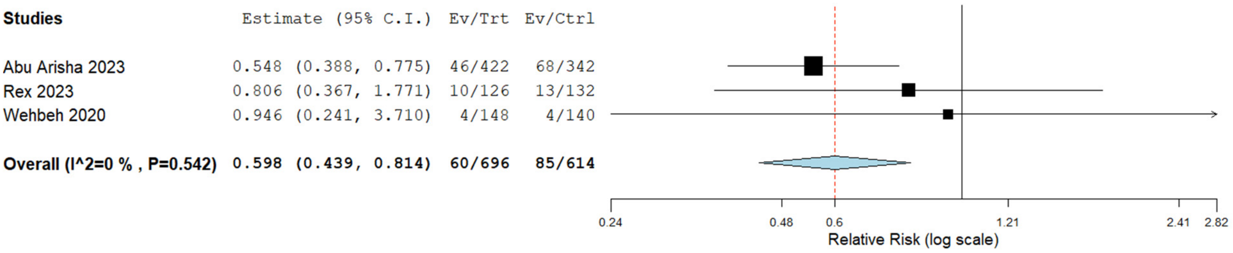

Supplementary Figure S1. A. Forest plot for recurrence rate in snare tip soft coagulation compared to placebo in studies. B. Forest plot demonstrating post polypectomy syndrome in snare tip soft coagulation compared to placebo in studies. C. Forest plot showing perforation rate in snare tip soft coagulation compared to placebo in studies. D. Forest plot for delayed bleeding in snare tip soft coagulation compared to placebo in studies. E. Forest plot demonstrating intraprocedural bleeding in snare tip soft coagulation compared to placebo in studies. F. Forest plot illustrating total adverse events in snare tip soft coagulation compared to placebo in studies in this meta-analysis.

A.

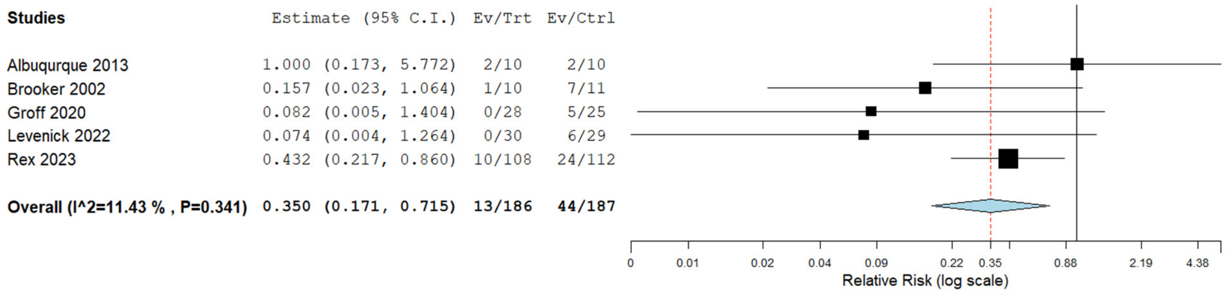
Studies
Estimate (95% C.I.) Ev/Trt Ev/Ctrl
Albuquerque 2013 1.000 (0.173, 5.772) 2/10 2/10
Brooker 2002 0.157 (0.023, 1.064) 1/10 7/11
Groff 2020 0.082 (0.005, 1.404) 0/28 5/25
Levenick 2022 0.074 (0.004, 1.264) 0/30 6/29
Rex 2023 0.432 (0.217, 0.860) 10/108 24/112
Overall (I^2=11.43 % , P=0.341) 0.350 (0.171, 0.715) 13/186 44/187
0 0.01 0.02 0.04 0.09 0.22 0.35 0.88 2.19 4.38
Relative Risk (log scale)

B.

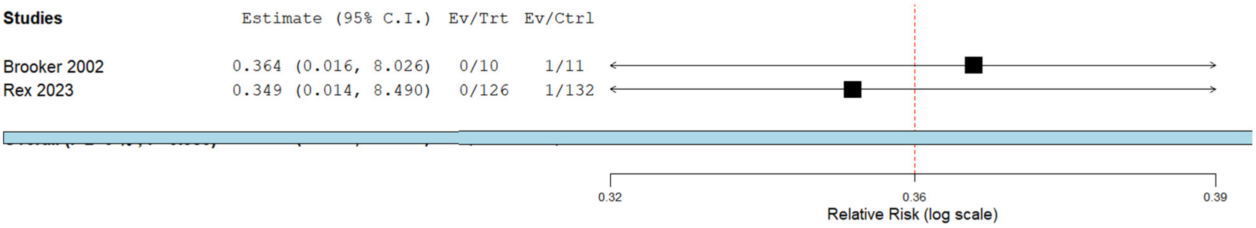
Studies
Estimate (95% C.I.) Ev/Trt Ev/Ctrl
Brooker 2002 0.364 (0.016, 8.026) 0/10 1/11
Rex 2023 0.349 (0.014, 8.490) 0/126 1/132
0.32 0.36 0.39
Relative Risk (log scale)

C.

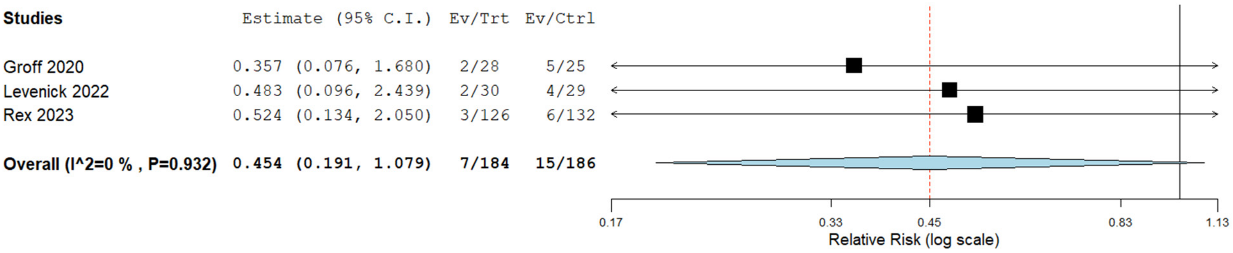
Studies
Estimate (95% C.I.) Ev/Trt Ev/Ctrl
Groff 2020 0.357 (0.076, 1.680) 2/28 5/25
Levenick 2022 0.483 (0.096, 2.439) 2/30 4/29
Rex 2023 0.524 (0.134, 2.050) 3/126 6/132
Overall (I^2=0 % , P=0.932) 0.454 (0.191, 1.079) 7/184 15/186
0.17 0.33 0.45 0.83 1.13
Relative Risk (log scale)

D.

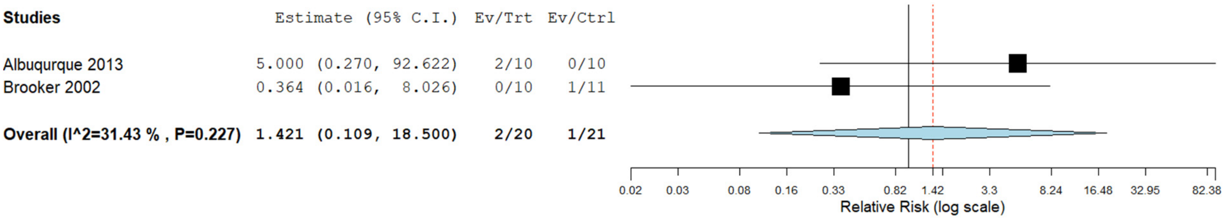
Studies
Estimate (95% C.I.) Ev/Trt Ev/Ctrl
Albuquerque 2013 5.000 (0.270, 92.622) 2/10 0/10
Brooker 2002 0.364 (0.016, 8.026) 0/10 1/11
Overall (I^2=31.43 % , P=0.227) 1.421 (0.109, 18.500) 2/20 1/21
0.02 0.03 0.08 0.16 0.33 0.82 1.42 3.3 8.24 16.48 32.95 82.38
Relative Risk (log scale)

E.

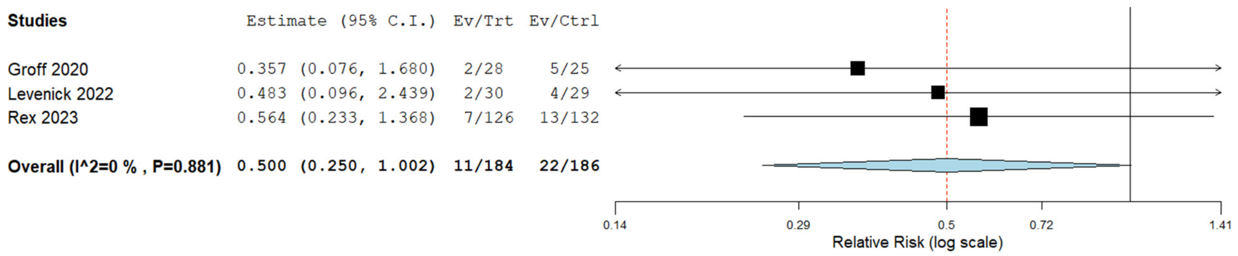

Supplementary Figure S2. A. Forest plot for recurrence rate in argon plasma coagulation compared to placebo in studies. B. Forest plot demonstrating post polypectomy syndrome in studies comparing argon plasma coagulation compared to placebo. C. Forest plot for delayed bleeding in studies comparing argon plasma coagulation to placebo. D. Forest plot demonstrating intraprocedural bleeding in studies comparing argon plasma coagulation to placebo. E. Forest plot illustrating total adverse events in studies comparing argon plasma coagulation to placebo in this meta-analysis.

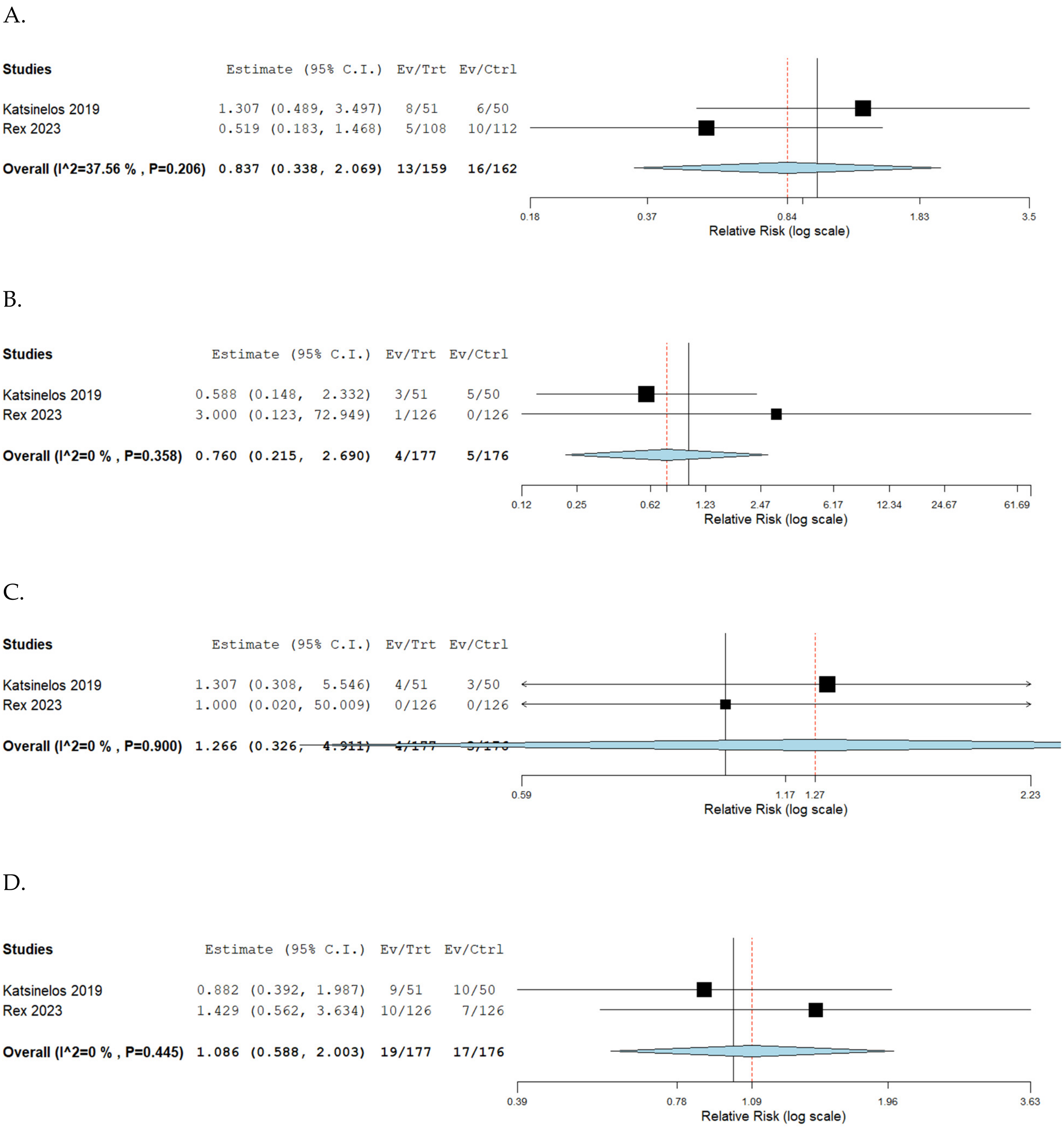

Supplementary Figure S3. A. Forest plot for recurrence rate in snare tip soft coagulation compared to argon plasma coagulation in studies. B. Forest plot demonstrating post polypectomy syndrome in snare tip soft coagulation compared to argon plasma coagulation in studies. C. Forest plot showing perforation rate in snare tip soft coagulation compared to argon plasma coagulation in studies. D. Forest plot illustrating total adverse events in snare tip soft coagulation compared to argon plasma coagulation in studies in this meta-analysis.

A.

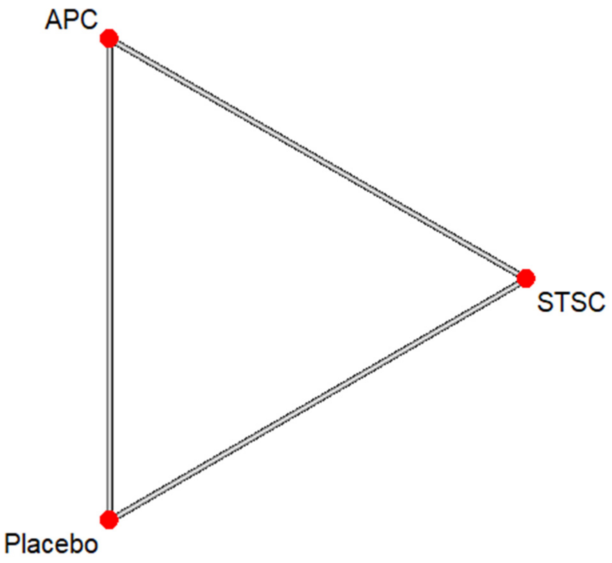

B.

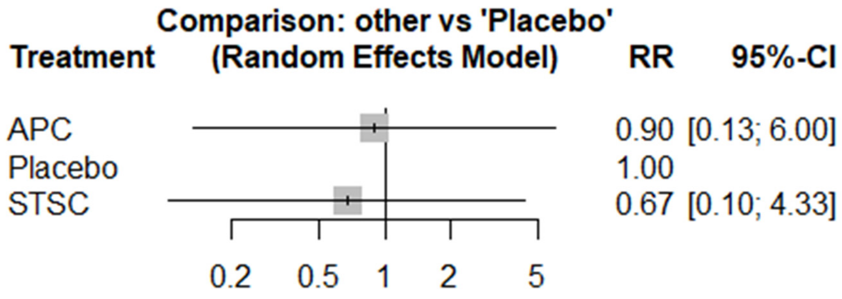

Supplementary Figure S4. Post polypectomy syndrome events using network meta-analysis: (A) Network diagram (the line represents a direct comparison in studies, and width of line represents number of studies), (B) Forest plot with Placebo as comparison group (APC: argon plasma coagulation, CI: confidence interval, RR: relative risk, STSC: snare tip soft coagulation).

A.

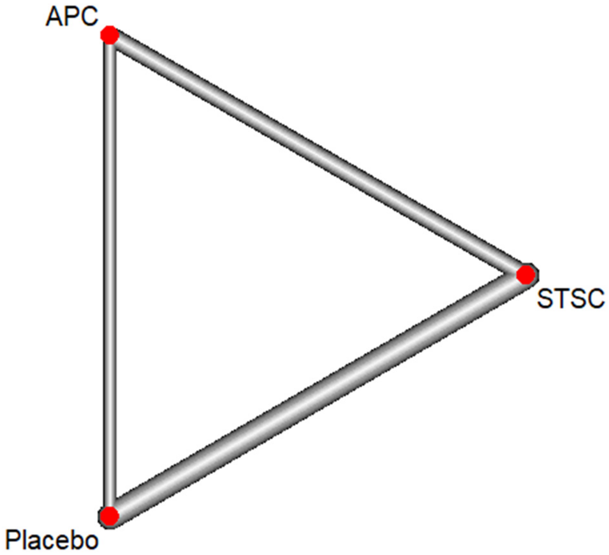

B.

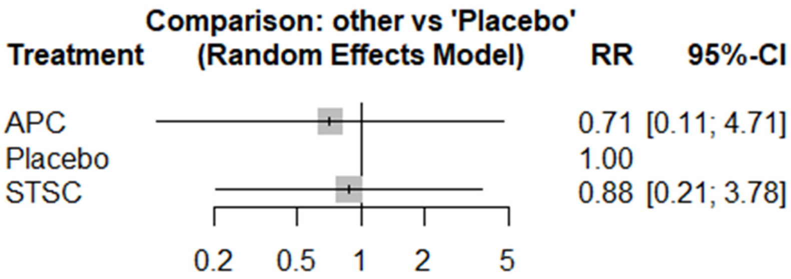

Supplementary Figure S5. Perforation events using network meta-analysis: (A) Network diagram (the line represents a direct comparison in studies, and width of line represents number of studies), (B) Forest plot with Placebo as comparison group (APC: argon plasma coagulation, CI: confidence interval, RR: relative risk, STSC: snare tip soft coagulation).

A.

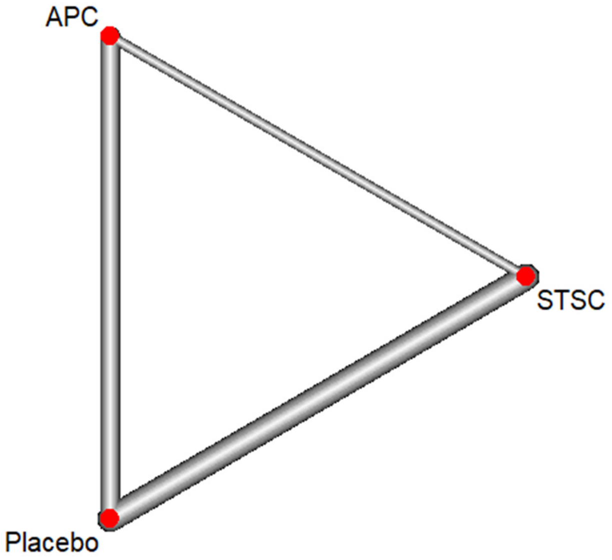

B.

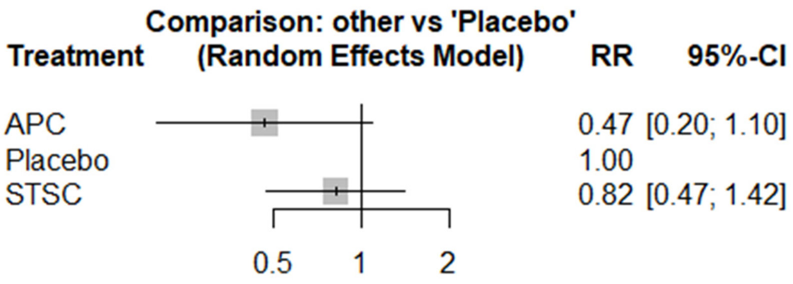

Supplementary Figure S6. Delayed bleeding events using network meta-analysis: (A) Network diagram (the line represents a direct comparison in studies, and width of line represents number of studies), (B) Forest plot with Placebo as comparison group (APC: argon plasma coagulation, CI: confidence interval, RR: relative risk, STSC: snare tip soft coagulation).

A.

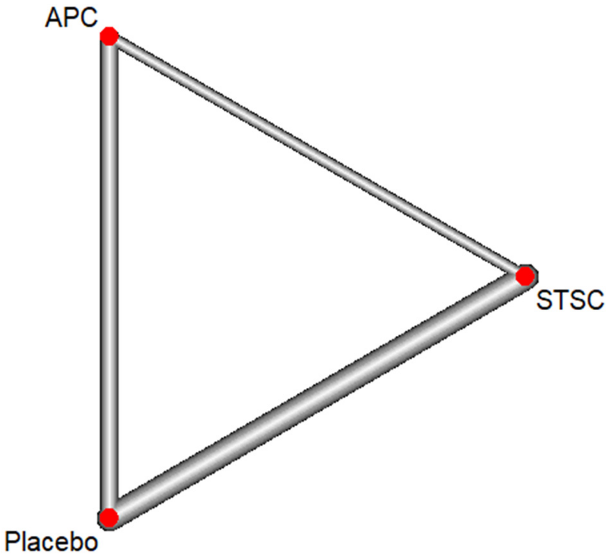

B.

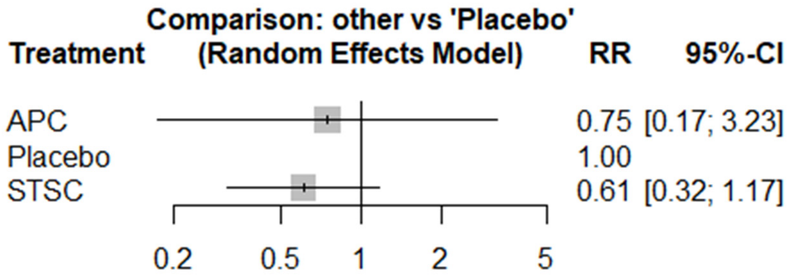

Supplementary Figure S7. Intraprocedural bleeding events using network meta-analysis: (A) Network diagram (the line represents a direct comparison in studies, and width of line represents number of studies), (B) Forest plot with Placebo as comparison group (APC: argon plasma coagulation, CI: confidence interval, RR: relative risk, STSC: snare tip soft coagulation).

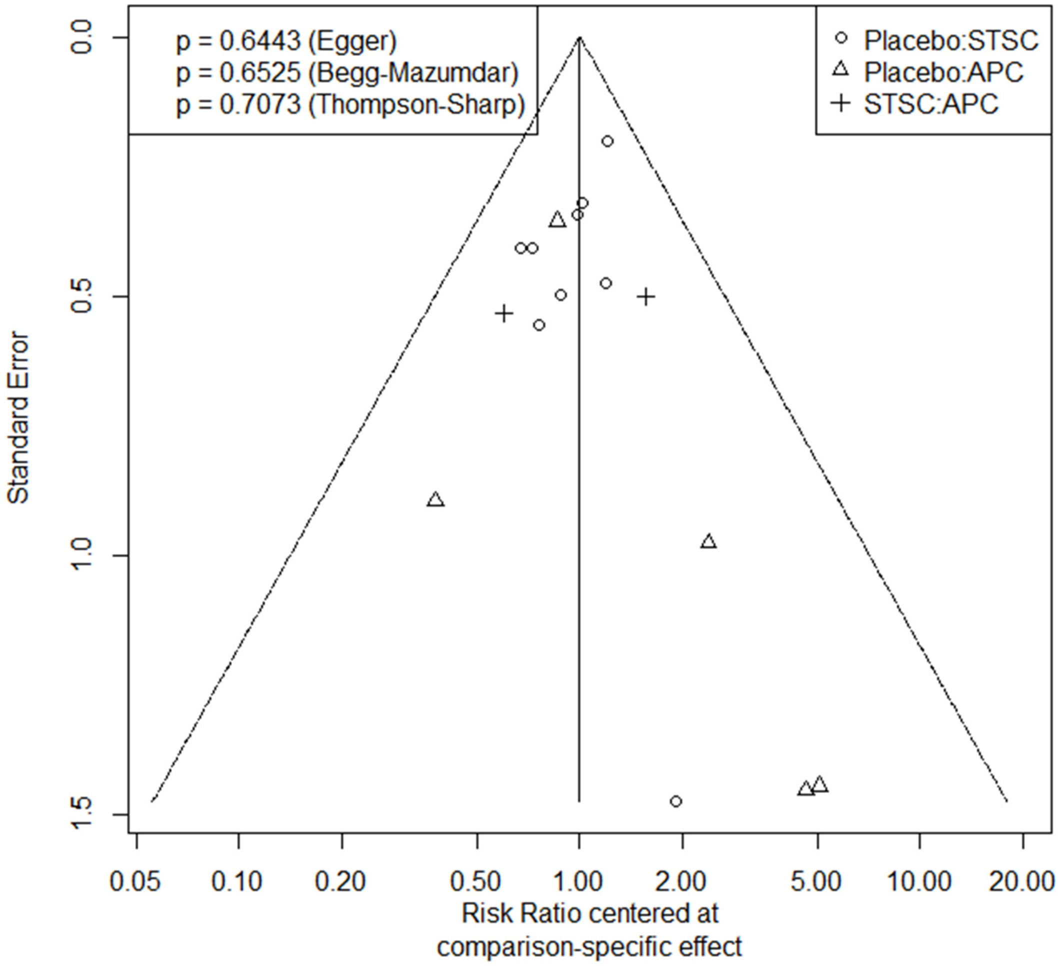

Supplementary Figure S8. Publication bias analysis using funnel plots and Egger's and Thompson–Sharp regression analysis (APC: argon plasma coagulation, STSC: snare tip soft coagulation).
